# Listening effort measurement by pupillometry under dual-task paradigms: the effect of auditory spectral resolution

**DOI:** 10.1007/s00405-025-10000-2

**Published:** 2026-02-16

**Authors:** Fahrettin Deniz Senli, Aysegul Ozkan, Cengiz Acarturk, Betul Cicek Cinar

**Affiliations:** 1 Department of Audiology, Yildirim Beyazit University, Ankara, Türkiye; 2 Department of Audiology, Hacettepe University, Ankara, Türkiye; 3 Department of Psychology, Başkent University, Ankara, Türkiye; 4 Center for Cognitive Science, Jagiellonian University, Kraków, Poland

**Keywords:** Cochlear implant, Dual-task, Listening effort, Pupillometry, Reaction time

## Abstract

**Purpose:**

This study evaluated the relationship between auditory spectral resolution and listening effort using cochlear implant simulations in 21 normal-hearing participants.

**Methods:**

In a dual-task paradigm, participants repeated noise-vocoded sentences to 4, 6, 8, and 12 channels, as well as everyday normal speech, while performing a secondary rhyme-judgment task. Listening effort was measured via secondary-task reaction time and pupil dilation.

**Results:**

Decreased spectral resolution increased both reaction time and pupil size, indicating greater effort. Reaction time increased significantly only in the most degraded conditions (4 and 6 channels), whereas pupil dilation increased across all degraded conditions compared to intact speech. Speech intelligibility, although affected by degradation, did not predict either of the effort measures. The weak correlation between reaction time and pupil size suggests they capture related but distinct aspects of listening effort.

**Conclusion:**

These findings highlight the multidimensional nature of listening effort, demonstrating that physiological measures can reveal increased cognitive load even when behavioral performance is unaffected. Combining these measures is crucial for a comprehensive assessment of the cognitive consequences of perceiving degraded speech.

## Introduction

Recent research on speech understanding in challenging listening conditions has focused on the perception of listening effort. Listening effort is generally understood as the allocation of cognitive resources to process auditory stimuli and achieve a listening goal, involving the intentional use of mental resources to overcome listening difficulties [1, 2].

Previous studies have shown that listening effort is influenced by multiple factors, including hearing loss, age, speech rate, background noise, and contextual cues that affect speech understanding [3]. Attention and working memory are considered primary cognitive resources in effortful listening. This study measures listening effort via behavioral (reaction time) and physiological (pupil size) responses to noise-vocoded speech, which simulates the reduced spectral resolution characteristic of cochlear implants (CIs). While not fully mimicking CI auditory processing, this method allows a controlled investigation of how spectral degradation impacts listening effort.

Cochlear implants are medical devices that convert sound into electrical signals to restore hearing for individuals with severe to profound hearing loss. While CIs preserve temporal envelope information, the resulting signal often lacks the fine spectral detail of natural hearing, leading to reduced spectral resolution. CI use has increased, with more patients receiving them in the past decade [4]. The primary goal of CIs is to provide audibility and speech understanding, traditionally assessed through speech intelligibility tests [5]. Although CIs significantly improve speech understanding in quiet [6], the transmitted signal’s reduced spectral and temporal resolution means it lacks the richness of natural hearing. This degradation can negatively impact speech perception in noise [7] and require significant cognitive effort for optimal intelligibility [8].

Various methods are used to measure listening effort, including subjective self-report questionnaires [9], behavioral measures [10, 11], and physiological measures [12]. This study employs a dual-task paradigm, with speech intelligibility as the primary task and a rhyme judgment task as the secondary task. This well-established behavioral method is rooted in the limited-capacity model of cognitive resources [13]. This model posits that individuals have limited cognitive resources. When performing tasks simultaneously, resources are primarily allocated to the main task. With increasing difficulty in primary task demands more resources, thus rusilts in impairing secondary task performance [14]. A key strength of this approach is its objective, indirect measure of cognitive load, avoiding self-report biases [1]. Dual-task paradigms have been effectively applied in various auditory and cognitive domains, showing sensitivity to changes in task difficulty and cognitive allocation [15]. This sensitivity to subtle changes in auditory signal quality makes them valuable for examining listening effort [16]. For instance, Pals et al., using CI simulations, found that while speech intelligibility improved with increasing spectral resolution, secondary task reaction times continued to decrease up to eight channels [17]. However, dual-task methods have limitations, such as potential inconsistencies due to task design variability [18]. Nonetheless, when carefully controlled, the dual-task paradigm remains an effective tool for understanding the relationship between auditory processing and cognitive effort [19].

Our study also employs pupillometry, a physiological measure of listening effort that is sensitive to auditory signal changes and higher-level processing, complementing the behavioral reaction time measure. Larger pupil size typically reflects increased attention and autonomic nervous system activity [20] and has been interpreted as an indicator of increased listening effort in complex auditory tasks [21]. Winn et al. investigated the relationship between spectral resolution and listening effort in individuals with hearing impairment using pupillary measures [22]. They reported pupil dilation with degraded spectral resolution, indicating greater effort, even with perfect intelligibility. Their study also compared pupillary responses to other cognitive tasks, providing a standard metric for comparison. These findings suggest that pupillometry offers a sensitive window into cognitive load during auditory tasks and can help understand the effects of signal degradation on listening effort in individuals with hearing impairment.

The present study investigates the relationship between spectral resolution and listening effort in CI-simulated speech. By integrating the dual-task paradigm and pupillometry, we aim to extend previous research [23, 24] and explore the effect of different languages in different phonetic contexts. Integrating both behavioral (reaction time, indexing processing speed and attentional allocation) and physiological (pupillometry, reflecting autonomic responses to cognitive load) measures offers a more comprehensive understanding of listening effort. We hypothesize that more challenging speech recognition (i.e., fewer spectral channels in noise-vocoded stimuli) will lead to increased pupil size and longer reaction times.

## Methods

The study was approved by the Applied Ethics Research Center of the Middle East Technical University with a protocol number of 232ODTU2020. Informed written consent was obtained from all participants involved in the study. All participants were fully informed of the study’s objectives and procedures prior to participation.

The target sample size was determined based on an interim power analysis conducted with G*Power (Version 3.1.9.1 [25] after data from 10 participants were collected. To achieve 80% statistical power at a significance level of α = 0.05 for detecting the expected differences in reaction times between key spectral conditions, this analysis indicated a required sample of approximately 17 participants.

### Participants

Twenty-one adults (14 females, 7 males) with normal hearing participated in this study (mean age, M = 24.66, SD = 0.47). The participants were native Turkish speakers, the language in which the stimuli were presented. They reported no language impairment or neurological or psychiatric disease. All participants reported right-hand dominance. The pure tone thresholds of all participants were measured with an Interacoustics AC40 audiometer. The pure tone thresholds were below or equal to 20 dB HL at audiometric frequencies ranging from 125 to 8000 Hz. Participants verbally confirmed that their vision was clear and free of blurriness. Those who required corrective lenses (glasses or contact lenses) were excluded from participation. Before asking for a voluntarily signed informed consent form, information about the task was provided. During the preprocessing of pupil size data, trials with a blink rate greater than 50% and trials that began or ended with a blink were eliminated. Data from one participant were excluded from the analyses due to the removal of 87,3% of the trials during data screening.

### Dual-task paradigm

The present study was designed to manipulate the demand for the primary task, which is based on speech intelligibility. The dual-task paradigm was employed to assess the participants’ listening effort, where the primary task was speech intelligibility with the CI-simulated speech. The secondary task was a rhyme judgment task involving visually presented pairs of monosyllabic words. The rhyme judgment task was selected as the secondary task because it taps into phonological processing, which is closely linked to auditory spectral resolution. When spectral degradation reduces phonetic detail, as in vocoded speech, phonological representations may become less precise, making rhyme judgments more cognitively demanding. This interaction enables us to investigate whether increased effort in decoding degraded auditory input affects the availability of cognitive resources for a simultaneous linguistic decision task. Previous studies have shown that phonological tasks such as rhyme judgments are sensitive to variations in auditory clarity and can reflect the allocation of cognitive resources under degraded listening conditions. Our goal was to measure the performance of the secondary task, the reaction time of the rhyme judgment task. The experiments were designed and implemented using Experiment Builder v.2.3.38, the native software for the EyeLink 1000 Plus eye tracking equipment, with a desktop mount from SR Research.

### Vocoded speech stimuli

Speech stimuli were obtained from sentences in the Turkish version of the Hearing in Noise Test (HINT) [26]. Turkish differs from English, which is the language used in most prior cochlear implant simulation studies, in several typological respects. One major difference is its agglutinative morphology. Turkish encodes multiple grammatical functions (such as tense, aspect, mood, and person) through the concatenation of suffixes onto a lexical root. For example, the verb gidiyorsun (“you are going”) consists of the root git- (“go”) followed by the progressive marker -iyor and the second person singular marker -sun. Turkish also allows subject omission (i.e., it is a pro-drop language), because verb morphology reliably indicates person and number. Moreover, although Turkish has a canonical subject–object–verb (SOV) word order, it exhibits considerable flexibility in word order, allowing pragmatic and discourse-related factors to influence it.

In contrast, English adheres more strictly to a subject–verb–object (SVO) order with limited variation. For instance, the sentence Çok hızlı gidiyorsun (“You are going very fast”) demonstrates both subject omission and morphological marking of tense and person on the verb. At the same time, Köpekleri dışarı çıkardık (“We took the dogs outside”) illustrates object fronting and verb-final placement. Additionally, Turkish has a shallow orthography, with a near one-to-one correspondence between graphemes and phonemes, which can facilitate phonological decoding [27].

The speech stimuli in the present study were continuous holistic sentential streams rather than being divided into utterances of single words. They were balanced in terms of phonetically and difficulty, semantically meaningful, and had the common grammatical order of the native language of the experiment. The stimuli were recorded by a professional male, native speaker. Each sentence was recorded in.wav sound format in a separate file and then normalized to 65 dB SPL root mean square (RMS) amplitude. The duration of the sentences ranged from 1.4 to 3.1 s (M = 2.31 s, SD = 0.514). The speech stimuli in our study were noise-vocoded using AngelSim™, a software tool designed to simulate the auditory experience of cochlear implant users [28]. The vocoder processes the speech signal by dividing it into a specified number of spectral channels (4, 6, 8, and 12 channels in this study), with each channel band-pass filtered using Greenwood’s filter function [29], which maps the frequency range of human hearing (200–7000 Hz) onto the cochlear implant intracochlear electrode array. The temporal envelope of the signal within each band is extracted and used to modulate a noise carrier, replacing the original signal in that band. This process reconstructs the speech signal with reduced spectral detail, simulating the limited spectral resolution experienced by CI users. By simulating the number of channels, AngelSim™ provides a controlled environment to study the effects of spectral degradation on speech perception and listening effort. The sentences were displayed at 8-second intervals. Since the average sentence duration was 2.31 s, a mean of approximately 5.69 s remained for the verbal responses of the participants. Each sentence was preceded by a 2 s silence before it began. Speech stimuli were presented monoaurally through Interacoustics TDH-39 headphones. To facilitate more appropriate statistical analysis, speech intelligibility scores were transformed into Rationalized Arcsine Units (RAU), which help stabilize variance and normalize the distribution of percentage scores, particularly when values approach floor or ceiling levels.

### Rhyme-judgement procedure

The words were monosyllabic, meaningful, and eligible for hearing-impaired studies [30]. Participants performed a rhyme judgment task, in which they determined whether two visually presented monosyllabic words rhymed. The word pairs were selected so that the probability of rhyme was 50%. If the words rhymed, the participant pressed “Z,” and if not, the participant pressed “P” on the QWERTY layout keyboard as fast as possible. All participants used the exact key mapping (‘Z’ for rhyme, ‘P’ for non-rhyme) and were instructed to use their left and right index fingers, respectively. Potential hand bias effects were statistically controlled by including the key pressed as a random effect in the linear mixed-effects models. The words were presented in black on a solid grey background without changing the brightness during the task. The word pairs were randomly presented on the screen for up to 2.5 seconds in each trial. After completing a trial, the next pair of words was displayed after a random interval of interstimulus (ISI) between 0.5 and 2.5 seconds (varied parametrically in intervals of 0.5 seconds). This ISI was included as the display of words periodically could affect reaction times. Participants would then pay attention before the display, resulting in an unrealistic short reaction time. In cases where there was no response from the participant, a “missing” label was recorded. Data cleaning involved the removal of incorrect responses and missing data. Furthermore, one trial was excluded for latency below 250 ms. These criteria resulted in the exclusion of 31 trials (1,73% of the total data). On average, 336 reactions per participant were recorded in the experimental sessions.

### Pupil recording

A nine-point calibration was used for eye movement recordings. The calibration was validated during the experiment session between the trials. Pupil sizes were recorded automatically from the right eye during the presentation of auditory stimuli using a temporal resolution of 1000 Hz and a typical spatial accuracy of 0.25°−0.50°. The brightness of the display background was kept constant during the experiment session since light conditions influence pupil size by stimulating the sympathetic nervous system [31]. The participants were instructed to keep their gaze on a visible dark grey box placed in the center of the screen to help with fixation. For preprocessing, blinks were first treated as artefacts. Blinks during recording were automatically detected by the EyeLink software. A window of 100ms before and 166ms after each blink was removed, and the resulting gaps were filled using linear interpolation. Following deblinking, a baseline correction was performed to account for trial-to-trial fluctuations in pupil size. We implemented a subtractive baseline correction, in which the mean pupil size during the first 50 milliseconds immediately following speech stimulus onset was subtracted from every sample within that trial. To reduce noise caused by blink-related artefacts and unstable baseline pupil measurements, we excluded trials with extreme baseline pupil sizes prior to analysis. Baseline pupil sizes were first converted to z-scores for each participant separately. Trials with z-scored baseline values exceeding ± 2 were identified as outliers and removed. Based on this criterion, 87 trials (4,38%) were excluded from the final dataset. The primary dependent variable was the average baseline-corrected pupil size for each trial, calculated over a time window from the stimulus onset to the offset of the auditory stimulus.

### Test procedure

The participants were seated in a quiet, soundproof room 60 cm away from the eye tracker. The eye tracker accurately recorded the participants’ eye movements and pupil sizes with high spatial and temporal resolution. A chinrest improves the quality of recorded gaze data.

Before the experiment, the participants received a briefing on the tasks and specific steps involved. Then, all participants completed a practice session to become familiar with the tasks. The practice session consisted of ten samples from each condition specified by the number of spectral channels. The primary task required participants to repeat the sentence presented as accurately as possible verbally. The secondary task, in this case, the rhyme judgment test, was also introduced during the practice session for a duration of three minutes. If participants found it challenging to perform the dual task, they were instructed to prioritize the primary task. This instruction would encourage them to allocate their limited resources more effectively. The participants performed the primary and secondary tasks simultaneously.

The participants performed the tasks under five different experimental conditions in a randomized order: a normal speech condition and others with noise-vocoded speech stimuli using different numbers of spectral channels (4, 6, 8, and 12). Each participant was presented with four lists for each test condition. Each list included 10 sentences, resulting in 40 sentences per condition. Participants completed two main experiments: a dual-task experiment and a pupillometry experiment. For each experiment, 100 sentences were presented, amounting to a total of 200 sentences per participant across the entire experiment. Auditory stimuli were presented to the right ear only to simulate unilateral CI users, using Interacoustics TDH-39 audiometric headphones at 65 dBA, a standard equipment used in previous research. The stimuli were presented in five blocks, in random order to avoid learning effects. Speech intelligibility (accuracy) scores were calculated per word by manually labelling the participant’s responses as true or false. The pupil data and the reaction times were obtained from Data Viewer v.4.2.1.

### Statistical analyses

Reaction times and pupil size were analyzed using two linear mixed models (LMMs) implemented with the *lmer*() function from the lme4 package in R [32, 33]. In the reaction time model, spectral channel condition was included as a fixed effect. To account for random variability, participants, word pairs, and response key (‘P’ or ‘Z’) were included as random effects. A natural logarithmic transformation was applied to reaction times to normalize the distribution. In the pupil size model, spectral channel condition and trial number were included as fixed effects, with participants included as a random effect. The full model parameters, including random effect variances and standard errors, are detailed in Tables 1 and Table 2. The final models’ p-values were computed using the *lmerTest* package [34]. Partial effects for visualization were extracted using the *remef* package, and all graphs were constructed using the *ggplot2* package [35]. A Pearson correlation test was used to calculate the correlation between response time and baseline-corrected pupil size results. Both variables were standardized using Z-scores prior to the analysis. Linear mixed-effects regression analyses were conducted for each of the five conditions to examine whether RAU-transformed speech intelligibility scores predict response time and pupil size. For all statistical tests, p-values below 0.05 were considered statistically significant.

**Table 1:** Summary of reaction time linear mixed model estimates

Predictor	Estimate (b)		Std. Error (SE)		df		t-value		*p*
Fixed Effects									
(Intercept)	6.70		0.03		9		198.86		< 0.001
4 Channels (4 C) *	0.08		0.02		422		5.01		< 0.001
6 Channels (6 C) *	0.05		0.02		429		3.25		0.001
8 Channels (8 C)	0.02		0.02		459		1.14		0.255
12 Channels (12 C)	0.02		0.01		3064		1.45		0.149
Random effects:									
Random Effects	Variance		Std.Dev. (SD)						
Word pairs (Intercept)	0.003		0.06						
Participant (Intercept)	0.011		0.11						
Key (Intercept)	0.001		0.03						
Residual	0.058		0.24						

*b* unstandardized regression coefficient, *SE* standard error, *df *degrees of freedom. Number of observations = 1761. Grouping levels: Word pairs = 312, Participant = 20, Key = 2

Annotation: * indicates that the effect of the variable is significant; a colon between the variables indicates the interaction

**Table 2:** Fixed effects estimates from the linear mixed model predicting pupil size

Predictor	Estimate (b)	Std. Error (SE)	df	t-value	*p*
Intercept (NS)	47.96	1.91	449.10	25.14	< 0.001
Trial Number	−0.018	0.028	1687.30	−0.64	0.524
12 Channels (12 C) *	26.78	2.09	1686.57	12.79	< 0.001
8 Channels (8 C) *	51.55	2.09	1683.88	24.68	< 0.001
6 Channels (6 C) *	82.50	2.11	1689.09	39.16	< 0.001
4 Channels (4 C) *	87.36	2.09	1687.61	41.89	< 0.001
Random Effects	Variance	Std.Dev. (SD)			
Participant (Intercept)	4.52	2.13			
Residual	734.56	27.10			
*b *unstandardized regression coefficient, *SE *standard error, *df* degrees of freedom. Number of observations = 1691. Grouping levels: Participant = 20. NS = Normal Speech					

Annotation: * indicates that the effect of the variable is significant; a colon between the variables indicates the interaction

## Results

### Reaction time

**Fig. 1:**
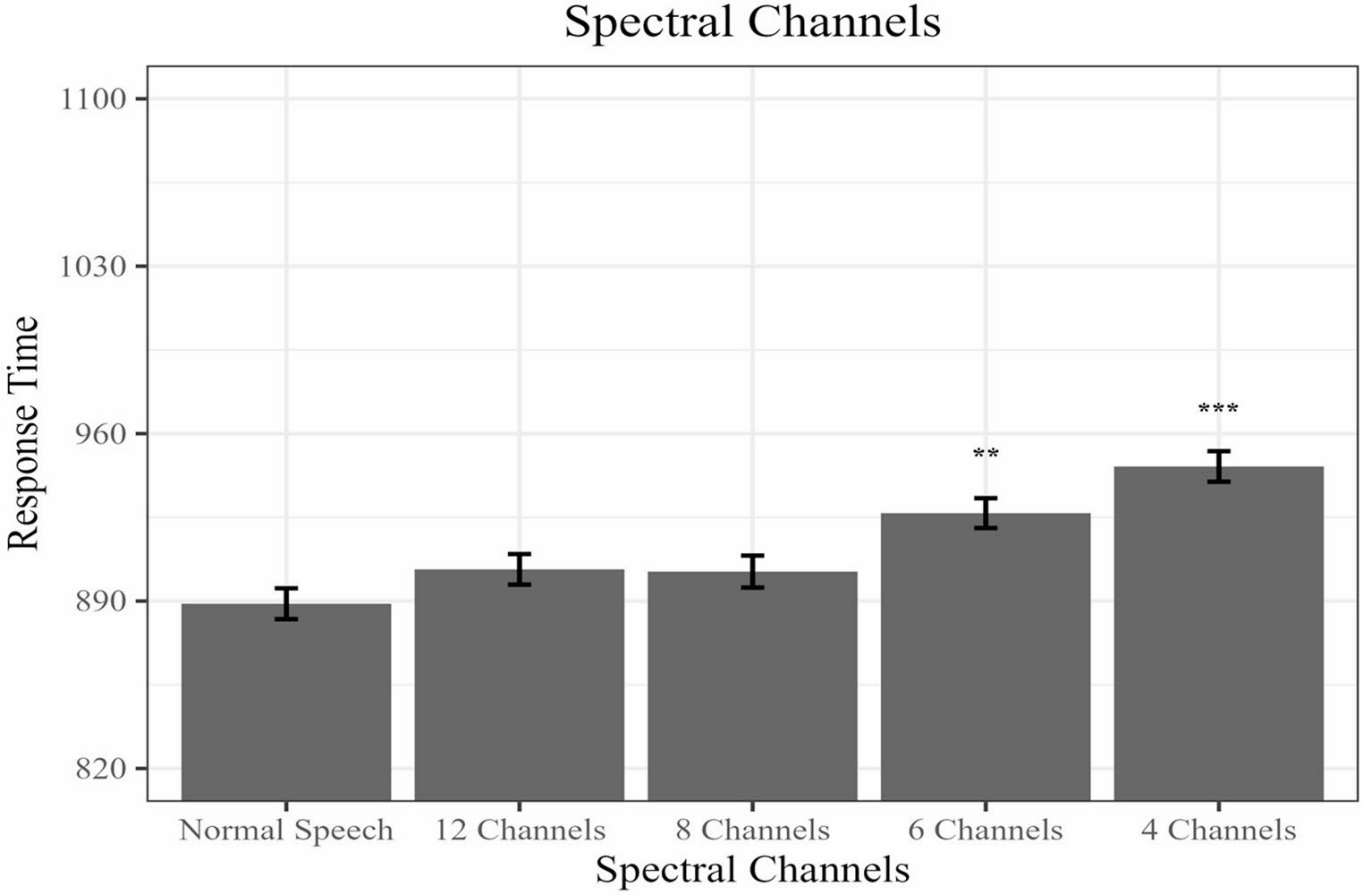
Model-estimated reaction times as a function of spectral channel conditions. The bars represent the mean partial effects derived from the linear mixed model, which controls random variability across participants, word pairs, and response keys. Error bars represent standard errors

A main effect was obtained for the reaction time (all *p*s < 0.001). Performance decreased significantly in the 4 channels (4 C) condition (*b* = 0.08, *SE* = 0.02, *t* = 5.01, *p* <.001) and 6 channels (6 C) condition (*b* = 0.05, *SE* = 0.02, *t* = 3.25, *p* <.01) when individuals reported rhyme judgment while simultaneously hearing vocoded speech, compared to normal speech. However, no significant differences were obtained in the conditions vocoded for 8 channels (*b* = 0.02, *SE* = 0.02, *t* = 1.14, *p* =.26) and 12 channels (*b* = 0.02, *SE* = 0.01, *t* = 1.45, *p* =.15) (Fig. 1).

### Pupil size

The model revealed a significant main effect of the listening condition, *F*(4, 1685.90) = 627.36, *p* <.001, while the effect of trial number was not significant, *F*(1, 1687.30) = 0.41, *p* =.524.

**Fig. 2:**
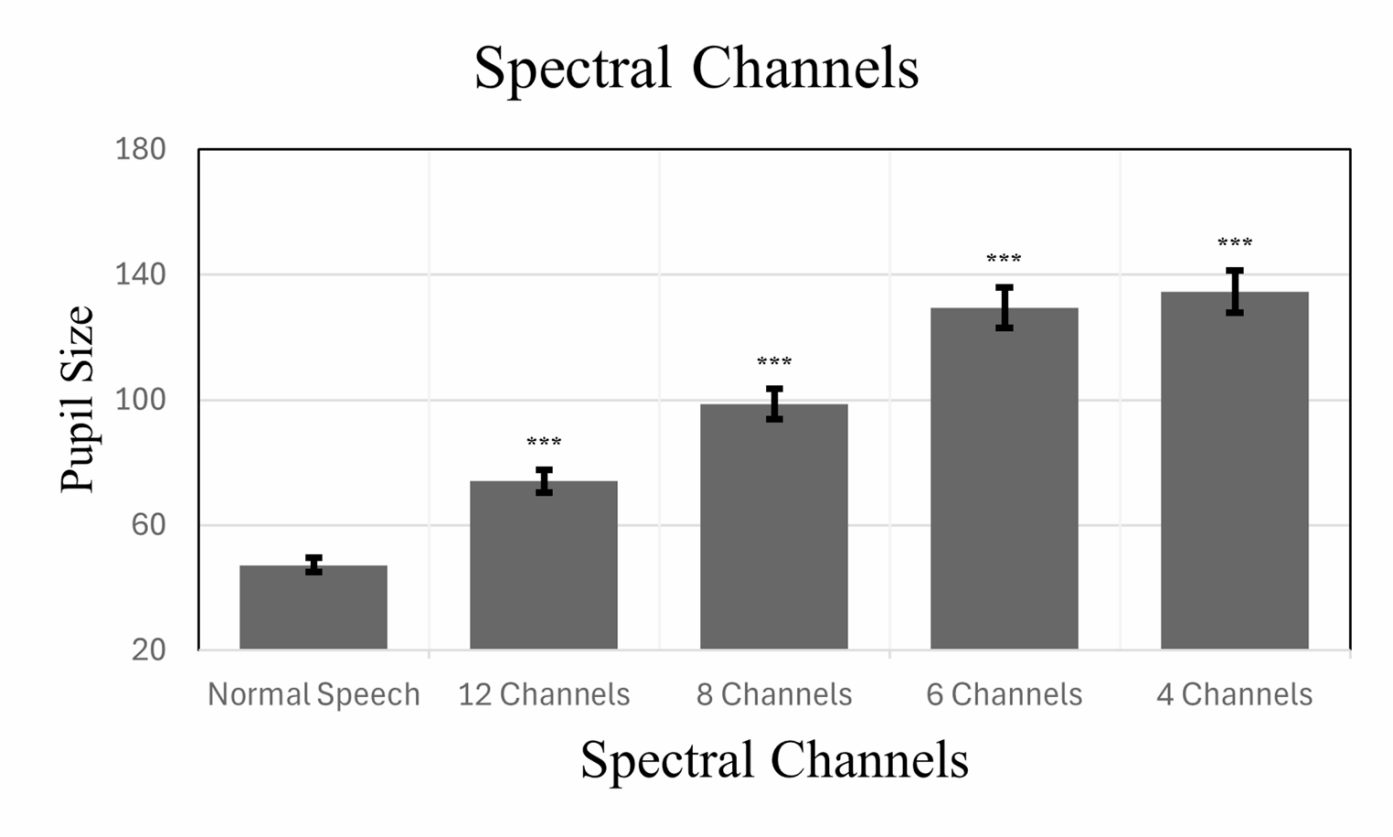
Model-estimated pupil size as a function of spectral channel conditions. The bars represent the fixed effects estimates derived from the linear mixed model, controlling for individual participant variability. Error bars represent standard errors

Fixed effects estimates showed that the number of channels decreased, the pupil size increased. Compared to the normal speech condition, pupil size was significantly larger in all vocoded conditions: 12 channels (*b* = 26.78, *SE* = 2.09, *t*(1686.57) = 12.79, *p* <.001), 8 channels (*b* = 51.55, *SE* = 2.09, *t*(1683.88) = 24.68, *p* <.001), 6 channels (*b* = 82.50, *SE* = 2.11, *t*(1689.09) = 39.16, *p* <.001), and 4 channels (*b* = 87.36, *SE* = 2.09, *t*(1687.61) = 41.89, *p* <.001). The trial number slope (*b* = − 0.018, *SE* = 0.028, *t*(1687.30) = − 0.64, *p* =.524) indicated no systematic change in pupil size over trials (Fig. 2).

### Speech intelligibility

The speech intelligibility scores were *M* = 99,8% (*SD* = 0,48) for normal speech, *M* = 98,2% (*SD* = 1,92) for 12 channels, *M* = 94,4% (*SD* = 4,76) for 8 channels, *M* = 93,1% (*SD* = 4,54) for 6 channels, and *M* = 88,8% (*SD* = 5,61) for 4 channels. The mean RAU-transformed speech intelligibility scores were *M* = 118,05 (*SD* = 4,82) for normal speech, *M* = 108,65 (*SD* = 6,52) for 12 channels, *M* = 100,6 (*SD* = 7,88) for 8 channels, *M* = 98,05 (*SD* = 9,19) for 6 channels, and *M* = 91,05 (*SD* = 5,8) for 4 channels conditions.

A one-way repeated measures ANOVA was performed to examine the effect of channel conditions on RAU-transformed speech intelligibility scores. Mauchly’s test indicated that the assumption of sphericity was met (W = 0.49, χ²(9) = 12.48, *p* =.190). There was a significant main effect of channel condition, F (4, 76) = 41.30, *p* <.001, η²_γ_ = 0.65 (large effect).

**Fig. 3:**
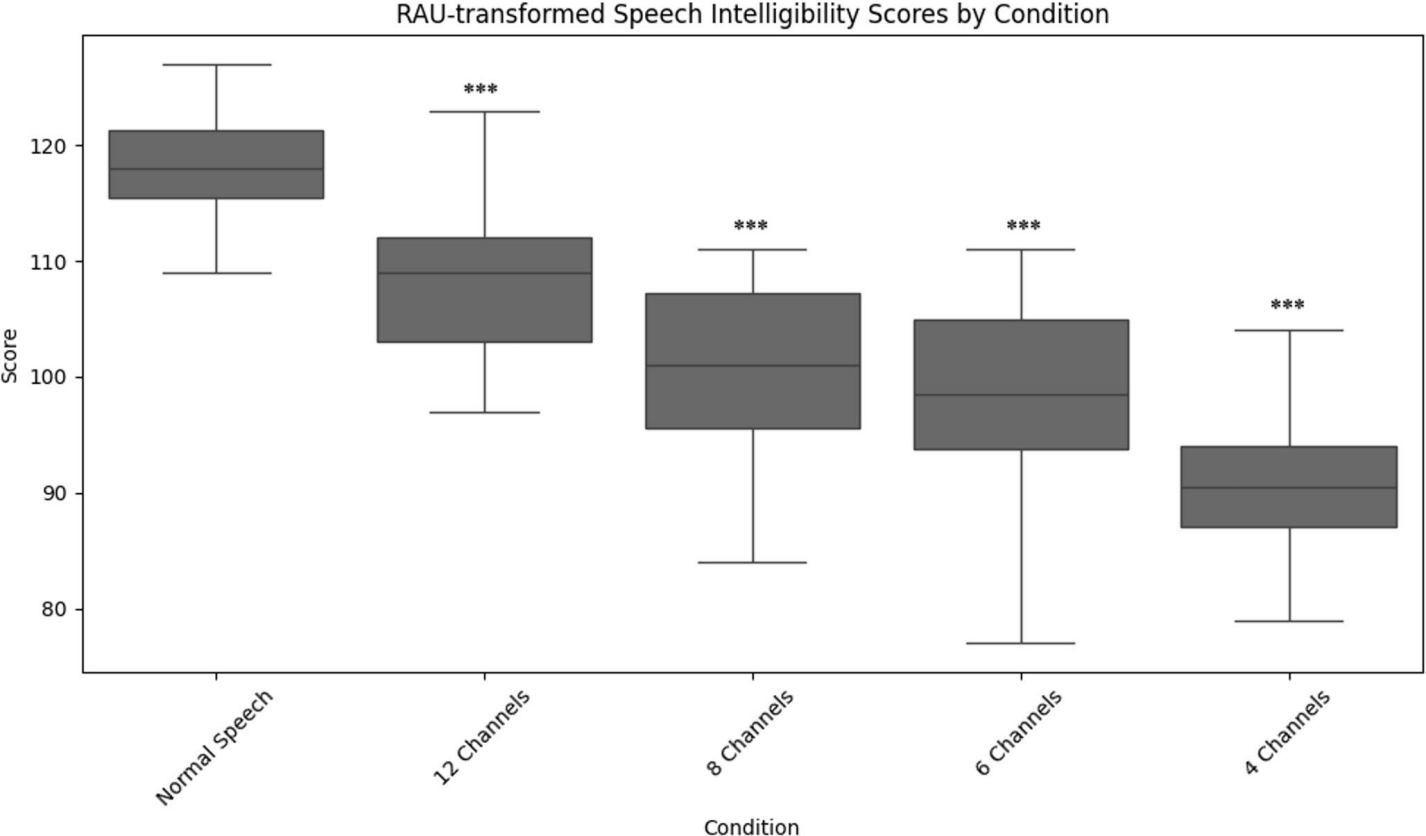
RAU-Transformed speech intelligibility scores for each channel. Illustrates the RAU-Transformed speech intelligibility scores for each channel

Post-hoc analyses with Bonferroni correction revealed that normal speech had significantly higher scores compared to 12 channels (*p* =.001, g = −1.60), 8 channels (*p* <.001, g = −2.61), 6 channels (*p* <.001, g = −2.66), and 4 channels (*p* <.001, g = −4.95) conditions (Fig. 3).

A series of linear mixed-effects regression analyses were conducted to examine whether RAU-transformed z-scored speech intelligibility scores predict z-scored response time and pupil size for five conditions. Participants’ codes were included as a random effect to account for within-subject variability. The results revealed no significant relationship between RAU-transformed z-scored speech intelligibility scores and z-scored response time in the normal speech (β = 0.082, SE = 0.058, df = 129.6, *p* =.290, *R²m* = 0.114, *R²c* = 0.224), 12 channels (β = −0.025, SE = 0.052, df = 132.1, *p* =.788, *R²m* = 0.157, *R²c* = 0.321), 8 channels (β = −0.087, SE = 0.064, df = 138.2, *p* =.184, *R²m* = 0.083, *R²c* = 0.158), 6 channels (β = −0.066, SE = 0.069, df = 139.4, *p* =.465, *R²m* = 0.149, *R²c* = 0.302), and 4 channels (β = −0.164, SE = 0.054, df = 128.2, *p* =.058, *R²m* = 0.152, *R²c* = 0.279) conditions.

For pupil size measurements, no significant relationship was found between RAU-transformed z-scored speech intelligibility scores and z-scored pupil size in the normal speech (β = 0.021, SE = 0.028, df = 120.4, *p* =.926, *R²m* = 0.889, *R²c* = 0.878), 12 channels (β = −0.116, SE = 0.031, df = 131.8, *p* =.598, *R²m* = 0.883, *R²c* = 0.853), 8 channels (β = −0.222, SE = 0.030, df = 120.9, *p* =.307, *R*²m = 0.911, *R*²c = 0.867), 6 channels (β = −0.251, SE = 0.034, df = 114.2, *p* =.246, *R²m* = 0.907, *R²c* = 0.841), and 4 channels (β = −0.139, SE = 0.026, df = 118.1, *p* =.526, *R²m* = 0.898, *R*²*c* = 0.886) conditions.

### Correlation between response time and pupil size

A Pearson correlation analysis was conducted to examine the relationship between standardized pupil size and standardized response time.

**Fig. 4:**
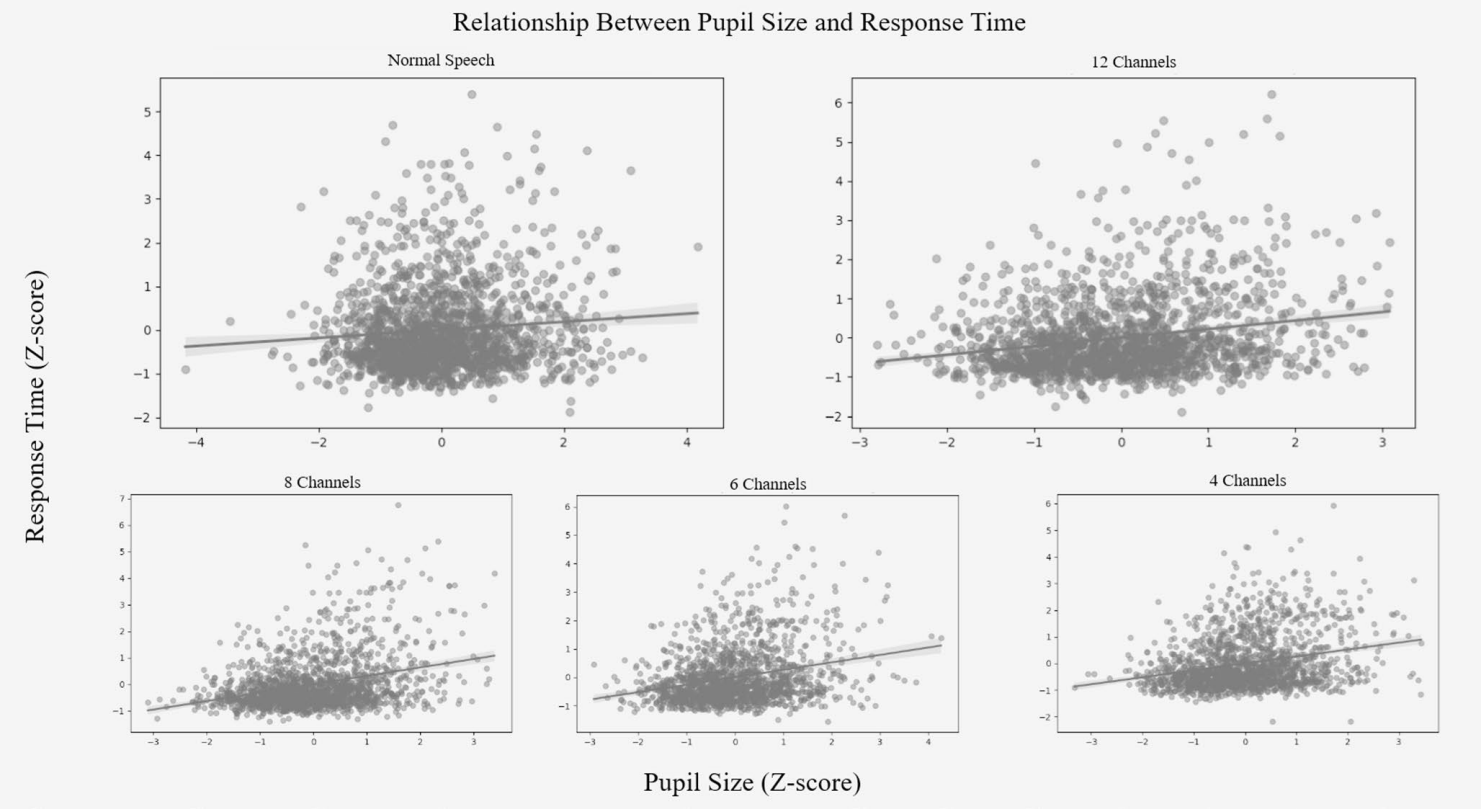
Correlation between z-scored response time and z-scored pupil size. Illustrates the correlation between z-scored response time and z-scored pupil size results

There were significant weak positive correlations for normal speech (*r*(1510) = 0.10, *p* <.001, *R*² = 0.011), 12 channels (*r*(1571) = 0.23, *p* <.001, *R*² = 0.059), 8 channels (*r*(1419) = 0.38, *p* <.001, *R*² = 0.121), 6 channels (*r*(1491) = 0.29, *p* <.001, *R*² = 0.068), and 4 channels (*r*(1472) = 0.28, *p* <.001, *R*² = 0.104) conditions (Fig. 4).

## Discussion

The present study employed a dual-task paradigm to investigate behavioral responses and pupillometry as physiological indicators of listening effort. The primary task involved speech intelligibility with simulated cochlear implant stimuli, while the secondary task required rhyme judgments on visually presented monosyllabic word pairs. Reaction times were recorded for the secondary task, and mean pupil size was measured with a high-resolution eye tracker. As spectral resolution decreased, both reaction time and pupil size increased across all spectral channel conditions. Significant but weak correlations were observed between response time and pupil size. RAU-transformed speech intelligibility scores did not independently predict reaction time or pupil size.

Reaction times differed significantly between normal speech and the 6-channel and 4-channel conditions. Increasing the number of spectral channels led to shorter reaction times, up to 6 channels. Listening effort decreased with increased spectral resolution, with a notable reduction observed up to 8 channels in dual-task simulations of CI use [17]. Recent data further indicate that CI users do not benefit from more than seven channels in terms of listening effort [23]. As speech degradation increased below 4 and 8 channels, secondary task performance declined [36]. The current findings align with these results, demonstrating decreased listening effort as measured by reaction times with increased spectral channels, though this pattern was not significant in the 8-channel condition. Limited trial numbers and Turkish’s shallow orthographic-phonological mapping may explain this exception.

Changes in pupil size reflected the impact of spectral resolution on listening effort. Pupil size increased significantly with fewer spectral channels, with significant differences between normal speech and all vocoded conditions. Pupil dilation is widely accepted as a proxy for cognitive effort [37, 38]. During the perception of degraded speech, pupil responses differentiated between conditions depending on the presence of feedback [36]. Without feedback, significant differences emerged between normal speech and both 8-channel and 4-channel vocoded speech. Pupil size also increased with decreasing spectral resolution in studies using 4, 8, 16, and 32 channels [24].

Another primary finding of this study is the divergence between behavioral (reaction time) and physiological (pupil size) indicators of listening effort, which suggests these measures capture distinct aspects of cognitive load. This dissociation is effectively explained by the Framework for Understanding Effortful Listening (FUEL), which conceptualizes listening effort as a multidimensional phenomenon shaped by task demands and individual cognitive resources [2]. According to this framework, physiological responses such as pupil dilation index the allocation of cognitive resources and attentional load, while behavioral measures such as reaction time reflect the residual cognitive capacity available for task execution. Our results align closely with this model: pupil dilation increased consistently with greater spectral degradation, whereas reaction times only declined when the task demands presumably overwhelmed the listener’s available resources [39–42].

Following the FUEL model, our data suggest that pupillometry is sensitive to the initial recruitment of cognitive resources, even when behavioral performance is successfully maintained. In the 8- and 12-channel conditions, for example, the larger pupil size indicates that listeners were already allocating more resources to compensate for the degraded input, thereby preserving secondary task performance. This reflects the recruitment of cognitive resources that maintains performance levels [43]. However, as degradation became more severe in the 4- and 6-channel conditions, this compensatory capacity was surpassed, resulting in observable performance declines in reaction time [14]. Our findings align with the view that concurrent multitasking may be governed by a serial procedural bottleneck rather than true parallel processing. According to the Threaded Cognition theory, while multiple tasks can be active as ‘threads,’ they must compete for a single, serial procedural resource to initiate execution [44]. Similarly, Bottleneck Theory suggests that attention is allocated to only one task at a time, necessitating rapid switching between the primary and secondary tasks [45]. In our study, the degraded spectral resolution likely increased the processing duration required by the primary auditory task within this bottleneck. Consequently, the secondary rhyme-judgment task was forced to wait longer for procedural resources, manifesting as the prolonged reaction times observed in the 4- and 6-channel conditions.

While our results demonstrate the value of a multi-measure approach, it is crucial to acknowledge that the nature of the secondary task itself can contribute to such dissociations. The current lack of standardization in dual-task paradigms complicates the interpretation and comparison of listening effort outcomes across studies [18, 46]. Factors such as task selection, interference, prioritization, and even individual motivation can further affect results [47]. Establishing standardized protocols would therefore be a significant step toward improving the consistency, reliability, and generalizability of findings in listening effort research, allowing for a clearer understanding of the distinct cognitive processes indexed by different measures [39–42].

The role of spectral resolution in speech intelligibility scores has been investigated in previous studies [48]. In this study, intelligibility scores declined with fewer spectral channels. However, no relationship emerged between RAU-transformed speech scores and either reaction time or pupil size. Similarly, speech intelligibility did not predict pupil responses in earlier work [22]. Findings from CI user studies indicate that resolving linguistic ambiguity contributes more to effort than simple error rates, emphasizing that effort extends beyond intelligibility alone [49]. Although speech intelligibility and listening effort are related, they remain distinct constructs. Ceiling effects in intelligibility, especially above 4 channels, may have obscured relationships with effort metrics. Matching intelligibility across conditions could help isolate effort-related effects. Future studies may benefit from adaptive speech intelligibility tests that adjust difficulty to individual performance levels, thus avoiding ceiling effects.

A possible factor contributing to the observed pattern of listening effort may lie in the linguistic characteristics of the stimuli. Unlike English, which was the dominant language in prior cochlear implant simulation research, Turkish has several typological features that may influence how degraded speech is processed. Turkish’s agglutinative morphology leads to longer and morphologically dense words, where multiple grammatical cues are embedded in single verbal forms. This may increase cognitive load under degraded auditory input, even when intelligibility remains high. For example, listeners must extract person, tense, and aspect from a vocoded verb like *gidiyorsun*, which may demand greater working memory and syntactic integration effort compared to morphologically simpler English equivalents. Moreover, Turkish allows subject omission and flexible word order, requiring listeners to rely more heavily on morphological cues rather than fixed syntactic positions. These properties may make speech parsing more sensitive to spectral degradation and could partly explain why pupil dilation increased even in conditions where reaction times did not differ significantly. Future research could compare languages with varying morphological complexity to better understand how typological features interact with auditory degradation in shaping listening effort.

It is known that both pupillometry and reaction time are sensitive to the challenges of listening with reduced spectral resolution. However, these two metrics have typically been investigated separately. By integrating both in our study, we were able to reveal that they possess divergent sensitivity to the cognitive load imposed by spectral degradation. Crucially, pupillometry detected effort increases even in mild degradation (12 channels), whereas RT only reflected severe deficits (4–6 channels). This dissociation underscores that listening effort comprises distinct autonomic (pupillometry) and resource-allocation (RT) dimensions, supporting the FUEL model’s multidimensional framework. Furthermore, our tests on Turkish, a morphologically rich, agglutinative language, suggest that linguistic typology modulates effort under degradation, a finding that extends generalizability beyond Indo-European languages. Our dual-method approach offers clinicians a toolkit to identify ‘hidden’ effort in high-performing CI users, advocating for fittings that optimize both intelligibility and cognitive efficiency.

Although vocoded speech simulates CI processing, this approach has limitations. Actual CI processors may employ different frequency filters, and spectral resolution may vary among users [50]. Channel interactions also influence spectral resolution outcomes [51]. Listening effort may be higher with vocoded speech compared to real CI users [22]. In future studies, the effect of spectral resolution on listening effort can be evaluated with CI users. Furthermore, it is important to acknowledge that performance in complex dual-task paradigms can be influenced by individual variables not controlled in this study. Factors such as socioeconomic status and sleep quality can act as potential confounders in the evaluation of higher cognitive functions. While our within-subject design and the use of random effects in the statistical models help mitigate individual variability, future research should aim to control or stratify for these potential neurophysiological and environmental confounders. Despite this study has some limitations, it is one of the rare studies that have been conducted using stimuli created by simulating a cochlear implant using the Turkish language. Moreover, these findings may inform audiological rehabilitation strategies and CI processor programming by highlighting the impact of spectral resolution on cognitive load. Incorporating dual-task paradigms and pupillometry into clinical assessment may help identify individuals who experience greater listening effort despite high speech intelligibility scores.

## Conclusions

This study examined the relationship between spectral resolution and listening effort using a dual-task paradigm and pupillometry. Reduced spectral resolution was associated with increased reaction times and pupil size, indicating elevated listening effort. Speech intelligibility scores did not independently predict these effort-related measures. The dissociation between behavioral and physiological indicators highlights the complexity of listening effort and supports the use of complementary methods. While consistent with prior findings on pupil size and effort, methodological and linguistic differences must be considered. Overall, these results offer insight into how spectral resolution affects listening effort and underscore the importance of multifactorial assessment strategies.
